# Racial Disparities in Triple Negative Breast Cancer: A Review of the Role of Biologic and Non-biologic Factors

**DOI:** 10.3389/fpubh.2020.576964

**Published:** 2020-12-22

**Authors:** Om Prakash, Fokhrul Hossain, Denise Danos, Adam Lassak, Richard Scribner, Lucio Miele

**Affiliations:** ^1^Louisiana Health Sciences Center, School of Medicine, New Orleans, LA, United States; ^2^Department of Public Health and Preventive Medicine, St. George's University, True Blue, Grenada

**Keywords:** triple negative breast cancer, racial disparities, African-American women, non-hispanic whites, socioeconomic status, obesity, body mass index, waist-hip ratio

## Abstract

Triple-negative breast cancer (TNBC) is an aggressive subtype of breast cancer that lacks expression of the estrogen receptor (ER), progesterone receptor (PR), and human epidermal growth factor receptor (HER2). TNBC constitutes about 15–30 percent of all diagnosed invasive breast cancer cases in the United States. African-American (AA) women have high prevalence of TNBC with worse clinical outcomes than European-American (EA) women. The contributing factors underlying racial disparities have been divided into two major categories based on whether they are related to lifestyle (non-biologic) or unrelated to lifestyle (biologic). Our objective in the present review article was to understand the potential interactions by which these risk factors intersect to drive the initiation and development of the disparities resulting in the aggressive TNBC subtypes in AA women more likely than in EA women. To reach our goal, we conducted literature searches using MEDLINE/PubMed to identify relevant articles published from 2005 to 2019 addressing breast cancer disparities primarily among AA and EA women in the United States. We found that disparities in TNBC may be attributed to racial differences in biological factors, such as tumor heterogeneity, population genetics, somatic genomic mutations, and increased expression of genes in AA breast tumors which have direct link to breast cancer. In addition, a large number of non-biologic factors, including socioeconomic deprivation adversities associated with poverty, social stress, unsafe neighborhoods, lack of healthcare access and pattern of reproductive factors, can promote comorbid diseases such as obesity and diabetes which may adversely contribute to the aggression of TNBC biology in AA women. Further, the biological risk factors directly linked to TNBC in AA women may potentially interact with non-biologic factors to promote a higher prevalence of TNBC, more aggressive biology, and poor survival. The relative contributions of the biologic and non-biologic factors and their potential interactions is essential to our understanding of disproportionately high burden and poor survival rates of AA women with TNBC.

## Introduction

Breast cancer (BC) is the most commonly diagnosed cancer and the second leading cause of cancer death among women in the United States [American Cancer Society. Cancer Facts and Figures 2017]. Approximately 268,600 women will be diagnosed with BC, and nearly 41,760 will die with this malignancy in 2019 [American Cancer Society. Cancer Facts and Figures 2019].

Breast cancer is a heterogeneous disease consisting of distinct biological subtypes with a range of clinical, pathological, molecular, and genetic features and differing therapeutic responses and outcomes including the Black-White disparities in outcome (1). These differences have been demonstrated by molecular classification based on the expression of estrogen receptor (ER), progesterone receptor (PR), and human epidermal growth factor receptor 2 (HER2). Using this approach, at least four intrinsic subtypes of breast cancer have been identified. These include luminal A [ER+ and/or PR+, HER2- and low Ki67 (<14%)], luminal B [ER+ and/or PR+, HER2-, and high Ki67 (>14%) or ER+ and/or PR+, HER2+], HER2 [ER-, PR-, HER2 amplification], triple negative [ER-, PR-, HER2-, basal markers, such as cytokeratin (CK) 5/6, CK 14, CK 17, and epidermal growth factor receptor]. While the terms basal-like subtype (characterized as ER^−^/PR^−^/HER2^−^, basal-markers^+^) and triple negative breast cancer (TNBC) have been used interchangeably in some studies, evidence has shown that although most TNBCs are basal-like, up to 20–30% of them are not; additionally, not all basal-like breast cancers are TNBCs (2–4).

A recent population study in the United States has described four molecular breast cancer subtypes as mentioned above, based on the expression of three tumor markers, namely estrogen receptor (ER), progesterone receptor (PR), and human epidermal growth factor receptor (HER2) (5, 6). Luminal A subtype constituted the majority (72.6%), TNBC 13%, luminal B 5% and HER2-enriched constituted 10% of all breast cancers diagnosed in 2011 (7). The presence of these four breast cancer subtypes varied notably with age and race. But we do not know whether it also varied with healthcare variables such as access to healthcare resources.

Prominent racial differences have been noted in the incidence of and mortality from breast cancer between African American (AA) and Non-Hispanic White (NHW) women. The 2017 CDC and NCI review of trends in population-based BC incidence and mortality rates in 1999–2014 by age and race in 2014 indicates that although the incidence rates are comparable for AA and NHW women for all ages and stages of diagnosis the mortality rates are very different (8). AA patients have an ~2-fold higher mortality incidence compared with the NHW women, resulting in a disproportionately higher (>65%) risk of death (United States Cancer Statistics: 1999–2014 Incidence and Mortality Web-based Report at: http://www.cdc.gov/USCS). In addition, a greater proportion of five-year breast cancer-specific survival rates are significantly lower in AAs (78.9%) compared with NHWs (88.6%) (9). The mortality disparity is especially noteworthy in light of the similar incidence rates of breast cancer among AA and NHW women.

The reasons underlying the health disparities in breast cancer outcome are multifactorial and complex. Many biological and non-biological factors are perceived to contribute to these disparities. Significant molecular differences have been identified in the biological properties between AA women and NH White women. The present review is undertaken to provide a comprehensive view of how these factors contribute to marked differences in age of onset, stage of presentation and survival between AA and NH White women and eventually the development and outcome of the TNBC disparity. An understanding of these factors and how do they contribute to disparities is critical in defining an in depth understanding of the marked differences in development, presentation, and outcome of breast cancer between Caucasian and AA women. To address inequities, we begin the article with a description of the pattern of TNBC disparities among AA and NH White women. Because obesity is a well-documented factor exerting a significant effect on the development of breast cancer, in the second section we addressed the potential link between obesity and TNBC in AA women. Several studies have suggested that tumor biology may contribute to the outcome disparities with TNBC in AA women. Therefore, in the third section of the article, we address the biological mechanisms of TNBC risk in AA women. There is increasing evidence that lower socioeconomic status disproportionately promotes aggressive biology in AA patients with TNBC. Thus, the fourth section encompasses the social determinants of TNBC risk in AA women. The article will provide comprehensive view of the relationship between biological and non-biological factors to facilitate our understanding of disparities in the risk of TNBC, and to guide future efforts to eliminate such disparities.

## Methods

### Search Criteria

Literature searches were conducted in MEDLINE/PubMed to identify relevant articles published from 2005 to 2019 addressing breast cancer disparities primarily among AA women compared to NHW women in the United States. The studies were selected based on the relevance of their full-text contents examining the nature and magnitude as well as the major risk factors associated with breast cancer disparities. When relevant to our review article, specific papers identified from the reference lists of published papers were also included. The combination of keywords- “biologic factors and breast cancer (BC) and African American (AA) women, non-biologic factors and BC and AA women, obesity and BC and AA women, triple negative breast cancer (TNBC) and AA women, Social determinants and BC and AA women, and socioeconomic status (SES) and BC and AA women" were used.

### TNBC and AA Women

The distribution of breast cancer subtypes by race/ethnicity is illustrated in Table 1. As shown, the distribution of race/ethnicity within each subgroup compared with other subgroups varied significantly. The majority of the NHW and AA women had the luminal A (ER/PR+HER2-) subtype; NHW women had the highest prevalence compared with AA women.

**Table 1:** Frequencies of breast cancer subtype according to race.

**Study (reference)**	**Tumor type**	**NHW number (%)**	**AA number (%)**
Vallega et al. (10)	Luminal A	296 (42.05)	27 (21.95)
	Luminal B	75 (10.65)	3 (2.44)
	HER2 type	19 (2.70)	4 (3.25)
	Triple negative	66 (9.38)	24 (19.51)
	Other N/A	248 (35.23)	65 (52.85)
Kwan et al. (11)	Luminal A	1,464 (75.3)	92 (59.4)
	Luminal B	215 (11.1)	14 (9.0)
	HER2 type	60 (3.1)	5 (3.2)
	Triple negative	204 (10.5)	44 (28.4)
Lund et al. (12)	ER/PR+HER2-	231 (64.3)	41 (38.7)
	ER/PR+HER2+	29 (7.9)	7 (6.1)
	ER/PR-HER2+	21 (6.0)	12 (8.7)
	Triple negative	79 (21.8)	56 (46.6)
Sineshaw et al. (13)	HR+HER2-	126,856 (66.8)	15,253 (51.7)
	HR+HER2+	16,896 (8.9)	2,948 (10.0)
	HR-HER2+	7,130 (3.8)	1,463 (5.0)
	Triple negative	19,375 (10.2)	6,231 (21.1)
	Other N/A	19,620 (10.3)	3,590 (12.2)
Stewart et al. (14)	Luminal A	243 (42.33)	16 (30.19)
	Luminal B	62 (10.80)	0 (0.00)
	HER2 type	17 (2.96)	3 (5.66)
	Triple negative	67 (11.67)	10 (18.87)
	Other N/A	185 (32.23)	24 (45.28)
Plasilova et al. (15)	HR+ HER2-	175,760 (74.8)	20,255 (59.6)
	HR+ HER2+	22,870 (9.7)	3,744 (11.0)
	HR- HER2+	9,669 (4.1)	1,904 (5.6)
	Triple-negative	26,783 (11.4)	8,067 (23.7)

*HR, hormone receptor; ER/PR, estrogen receptor/progesterone receptor; HER2, human epidermal growth factor receptor 2; Luminal A, ER+ and/or PR+ HER2−; Luminal B, ER+ and/or PR+ HER2+; Triple negative, ER/PR− HER2−; N/A, not available*.

Luminal B (ER/PR+HER2+) and HER2-overexpressing subtype was least common among both races/ethnicities. Compared with NHW women, AA women are twice as likely to be diagnosed with TNBC indicating a disproportionate burden of TNBC in this population (10–15). Highly aggressive features of the TNBCs, lack of viable therapeutic targets and earlier age at onset, may contribute to poor outcomes and explain in part the poorest survival observed among AA women (12). In most studies listed in Table 1, the most common subtype was luminal A (ER+/PR+/HER2-), followed by TNBC, with luminal B and HER2+ expressing subtypes being less common. Luminal A subtype generally has the most favorable prognosis, whereas TNBC subtype has the least favorable prognosis (12, 16–19).

Compared with the luminal A subtype, TNBC is disproportionately more common in younger or premenopausal women, especially young AA women (12, 20, 21). Many studies show that relative to women of European decent premenopausal AA and African women have a high prevalence of TNBC (22–25). The Carolina Breast Cancer Study showed that the prevalence of the basal-like subtype of TNBC (39%; 38/97 invasive cancers) was substantially higher in premenopausal AA women than the prevalence of TNBC observed in post-menopausal AA women (14%; 14/99 invasive cancers) or American women of European descent (16%; 48/300 invasive cancers) (*p* < 0.001 for both comparisons) (26). The high frequency of TNBC in premenopausal AA women has been also observed in several subsequent population-based studies (12, 27–29). TNBC is biologically more aggressive and has poor prognosis than the luminal A subtype (18, 30). In addition, the 5 year relative survival rates for AA women diagnosed with breast cancer (80%) is significantly lower than for NH White women (91%) across all ages and tumor stages and subtypes, and age-adjusted mortality rate for AA women (30.0/100,000) is the highest rate for any ethnic group studied [Cancer Statistics 2017 (31)].

The racial differences in TNBC strongly persisted in all age categories with steep increase in TNBC incidence with increasing age for AA women compared with NHW women (21). As shown, the TNBC incidence rates were ~2-fold higher for AA women compared to NHW women in all age categories with highest TNBC incidence rate in the 60–74 year-old category (21). TNBCs most frequently occur in women with germline BRCA1 mutations (24). However, <20–25% of AA women with TNBC have a BRCA1 germline mutation. These data suggest that the molecular events surrounding TNBC initiation in AA women may be distinct from those of non-African descent.

### Obesity and TNBC in AA Women

Obesity is associated with increased risk of a variety of different cancer types. Of these obesity-associated cancer types, almost 13% of the cases worldwide, and nearly 20% of the cases in Europe and North America, are attributable to obesity (32, 33). In the United States, obesity rates have reached epidemic proportions. More than 60% of the adult US population falls in the overweight and obese categories as determined by BMI: 25–29.9 and >30 kg/m^2^, respectively (7, 34, 35). Results from the National Health and Nutrition Examination Survey (NHANES) have shown that obesity prevalence varied by sex, age, race, and socioeconomic status (25, 34–36). The recent 2-year NHANES survey showed that in the United States, 57.2% of AA women were obese vs. 38.2% NHW women (36). The overall prevalence of class 3 obesity (BMI > 40) in AA women was 16.8 vs. 9.7% in NHW women (36). Analyses of NHANES data from 2013 to 2014 showed that in addition to race, obesity and class 3 obesity prevalence also varied by age (36). As shown, the prevalence of obesity in AA women vs. NHW women was on an average 150% higher at all age groups, and the prevalence of class 3 obesity from ages 20–59 was 160% higher but at age ≥60 it was 240% higher (36).

A number of studies have investigated the potential link between obesity and breast cancer. In premenopausal women at high risk for breast cancer as defined by the Gail score, risk of invasive breast cancer was significantly increased in overweight and obese women compared to women of BMI < 25 kg/m^2^ (37). A recent study by Chen et al., with a large cohort of predominantly NH White women suggests a heterogeneity in the relationship between BMI and breast cancer molecular subtype risk (38). Compared to women with BMI < 25 kg/m^2^, obese premenopausal women (BMI ≥ 30 kg/m^2^) had an 82% higher risk of TNBC, and those in the highest weight quartile (quartiles were classified according to the distribution among luminal A patients) had a 79% increased risk of TNBC compared to those in the lowest quartile. Among post-menopausal women, obesity was associated with reduced risk of TNBC (Table 2). While other studies have suggested an increased risk of TNBC in premenopausal women associated with higher BMI, no differences in risk were found among postmenopausal women (11, 39, 40). Another study performed in West Virginia, the only state in Appalachia with a 95% White population, the TNBC incidence increased with increasing BMI. In this study, TNBC tumors were found to be significantly more common in those patients who were classified as obese, 49.6 vs. 35.8%, respectively (*P* = 0.0098) (41).The majority of studies done in North America and Europe found that the prevalence of TNBC in NHW women with breast cancer ranged from ~10–13% (13–15) which contrasts with the 18.9% seen in the West Virginian patients (41). The study of Gershuni et al. (7), showed association between BMI and TNBC in Black women but no association in non-Black women. The prevalence of TNBC in overweight and obese Black women was 2-fold compared to that in normal weight Black women. On the other hand, the incidence of TNBC was not significantly different among normal weight, overweight, and obese non-Black women. Stead et al. (28) found that the prevalence of TNBC in AA women was 3-fold higher as compared with non-Black women (Table 2) (28). However, stratifying the dataset to Black vs. non-Black women, they showed that, 29% of obese Black women had triple-negative tumors compared with 8.6% of obese non-Black women [odds ratio (OR) = 4.3: 95 CI = 1.8–10; *p* = 0.0004]. (Applying White/Caucasian women as the reference category, the OR = 4.2 and 95% CI = 1.6–13). Similarly, 31% of non-obese Black women had triple-negative tumors compared with 15% of non-obese non-Black women (OR = 2.7, 95% CI = 1.4–5.3; *p* = 0.003). (Applying White/Caucasians women as the reference category, the OR = 2.5 and 95% CI = 1.2–5.4) (Table 2). These two ORs did not differ significantly from each other (*p* = 0.41), indicating that BMI does not seem to be correlated with triple-negative status among AA women (28). Pierobon and Frankenfeld (42) used meta-analysis to sum up the results of 11 epidemiologic studies published between May 2008 and February 2012 to evaluate the association between obesity (BMI) and risk of TNBC. There results, in a case-case comparison of TNBC, showed that obese premenopausal women had 43% greater risk of TNBC than non-obese premenopausal women, but that obesity did not correlate with risk of TNBC in postmenopausal women. The relationship between obesity and TNBC remains uncertain, except that chronic inflammatory conditions induced by obesity may activate molecular pathways that favor TNBC pathogenesis.

**Table 2:** Body mass index and triple-negative breast cancer risk.

	**Pre-menopausal**		**Post-menopausal**	
**Chen et al. (38)**	***n* (%)**	**OR (95% CI)**	***n* (%)**	**OR (95% CI)**
**BMI (kg/m**^**2**^**)**				
<25	213 (37.1)	1.00 (ref)	216 (31.6)	1.00 (ref)
25–29.9	161 (28.0)	1.27 (0.92–1.75)	213 (31.2)	0.93 (0.67–1.29)
≥30	200 (34.8)	1.82 (1.32–2.51)	254 (37.2)	0.74 (0.54–1.00)
	**Non-Black**		**Black**	
**Stead et al**. **(****28****)**	**n/total (%)**	**OR (95% CI)**	**n/total (%)**	**OR (95% CI)**
**BMI (kg/m**^**2**^**)**				
<30	20/137 (15)	1	25/79 (32)	2.7 (1.4–5.3)
≥ 30	8/93 (8.6)	1	27/94 (29)	4.3 (1.8–10)

*BMI, Body mass index; OR, Odds ratio; CI, Confidence interval; n, Number*.

#### Racial Differences in Visceral Adipose Tissue and TNBC

BMI is usually associated with general obesity and does not provide any information on body composition. Waist circumference (WC) and waist-hip ratio (WHR), on the other hand, have been used as measures of central or intra-abdominal obesity, defined as WHR above 0.90 for males and above 0.85 for females (43). A number of studies have shown measurement of WC or WHR to be the best anthropometric indicator of visceral adiposity which is a key component of the metabolic syndrome associated with hyperinsulinemia and insulin resistance (44–46). The correlations between excess adiposity and TNBC are stronger in AA women than in NHW women. In a population-based case controlled Carolina Breast Cancer epidemiological study of AA and NHW women, the WHR was compared between the highest (≥0.84) and lowest (<0.77) groups in relation to the TNBC. Across all women, premenopausal women and postmenopausal women with high WHR had a significantly increased risk of developing TNBC compared with the lowest WHR group (25). The prevalence TNBC and TNBC risk factors was the highest among premenopausal African American women (39). There was no significant association between increased BMI (BMI ≥30 kg/m^2^) and the risk of TNBC (25). The study suggested potential association between WHR and TNBC and a lack of association between WHR and increased BMI.

Several other studies have also reported WHR as a strong risk factor for TNBC in AA women (39, 47–49). As shown in Table 3, Bandera et al., reported that recent BMI was not significantly associated with premenopausal TNBC (9). For postmenopausal women, high recent BMI was associated with reduced risk of TNBC. On the other hand, higher WHR (Table 3) was associated with non-significant increases of TNBC among premenopausal women. Among postmenopausal women, higher WHR was a stronger risk for TNBC for third and fourth quartiles, compared to lowest (48). The studies by Chollet-Hinton et al. (49) and Harris et al. (47) also suggested that abdominal adiposity as represented by WHR is an important factor contributing to young-onset disease. Higher WHR was more strongly associated with young (<40 years) premenopausal women at breast cancer diagnosis compared with older age (≥40 years) at diagnosis (Table 3) (49). As shown, ORs for BMI were not significantly modified by age, though obese BMI (≥30 kg/m^2^) was more strongly associated with a reduced association among young women. The study by Harris et al. (47) indicated that WHR was more strongly associated with an increased risk of premenopausal ER-negative breast cancer than risk of ER-positive breast cancer suggesting that sex hormone-independent pathways are involved in the higher risk of ER-negative breast cancer.

**Table 3:** Breast cancer incidence by BMI and WHR.

**(A) Bandera et al**. **(****48****)**								
	**Pre-menopausal**				**Post-menopausal**			
	**Cases**	**Controls**	**OR**	**95% CI**	**Cases**	**Controls**	**OR**	**95% CI**
**BMI (kg/m**^****2****^**)**								
<25	47	1,185	Ref		60	1,713	Ref	
25–29.99	73	1,253	1.29	0.85–1.94	71	2,874	0.55	0.37–0.84
30–34.99	56	814	1.43	0.91–2.23	77	1,926	0.72	0.47–1.08
≥35	51	835	1.13	0.71–1.80	56	1,460	0.6	0.39–0.93
**WHR**								
≤ 0.74	33	1,006	Ref		21	1,071	Ref	
0.75–0.81	55	938	1.26	0.78–2.03	40	1,106	1.33	0.76–2.31
0.82–0.88	63	851	1.18	0.73–1.91	72	1,232	1.73	1.02–2.91
>0.88	71	853	1.4	0.85–2.31	81	1,212	1.6	0.94–2.73
**(B) Chollet-Hilton et al. (49)**								
	**<40 years**				**≥40 years**			
	**Cases**	**Controls**	**OR**	**95% CI**	**Cases**	**Controls**	**OR**	**95% CI**
**BMI (kg/m^2^)**								
<25.0	149	466	1		283	955	1	
25–29.9	119	368	0.92	0.66–1.28	376	1,172	0.99	0.81–1.21
≥30.0	156	497	0.71	0.51–0.98	491	1,628	0.91	0.75–1.10
**WHR**								
<0.77	104	441	1		1,166	1,166	1	
0.77–0.83	128	341	1.14	0.81–1.59	952	952	1.02	0.83–1.25
≥0.84	163	394	1.46	1.04–2.05	1,285	1,285	1.11	0.91–1.35

*(A) TNBC in pre- and post-menopausal women. (B) Breast cancer incidence by age. BMI, Body mass index; WHR, Waist-hip ratio; OR, Odds ratio; CI, Confidence interval*.

#### Obesity and TNBC Development

Obesity may increase the incidence of TNBC in African and AA women through various biological mechanisms [reviewed in Chen et al. (38)]. In particular, centrally located adipose tissue creates a variety of physiological conditions that favor inflammation within the body. Dysregulation of certain biological functions such as cellular growth, angiogenesis stimulation, and extracellular matrix remodeling that favor tumor progression and relapse have been shown to result by adipokine secretion from adipose tissue. In addition, adipose tissue promotes an inflammatory response through the secretion of inflammatory mediators, such as tumor necrosis factor alpha (TNFα), interleukin 6 (IL-6), and retinol-binding protein-4 (RBP4). Studies have shown that TNFα promotes the growth of human breast cancer cells through the activation of several intracellular molecular pathways including NF-kappa B, MAP kinase and the PI3-K/AKT signaling pathways (50). Higher levels of circulating IL-6 is significantly associated with poor survival in patients with metastatic breast cancer (51, 52). IL-6 is also prognostic of early tumor size, tumor progression, and metastasis at various sites (53, 54).

Leptin is another cytokine predominantly produced by adipose tissue and is necessary for the development of normal breast gland and lactation. Serum leptin is significantly higher in obese than in normal weight individuals (55). This unusually high levels of leptin from excess adipose tissue may activate signaling that has been shown to induce relevant molecular pathways involved in proliferation, angiogenesis, and insulin-like growth factor 1 (IGF 1) expression resulting in tumor progression, invasion and metastasis (56, 57). On the contrary, adiponectin also secreted by adipose tissue, functions to block the tumorigenic effects of leptin. However, its expression is decreased in obese individuals and in those who are insulin resistant. This leads to an increased leptin to adiponectin ratio resulting in tumor proliferation in breast cancer cells (58). It has been shown that serum adiponectin is inversely related to breast cancer risk (59–61).

Several lines of evidence indicate a role for adiponectin in the pathways connecting insulin sensitivity to muscle morphology. Skeletal muscle is a heterogenous tissue comprising different fiber types which vary in their metabolic characteristic, that may play a role in the pathogenesis of obesity, hypertension, and diabetes. On the basis of these characteristics, muscle fiber are classified in to type I and type II fibers with type II further subdivided into type IIa and type IIb also known as type IIx (62, 63). Type I muscle fibers possess greater aerobic metabolic capacity because of higher myoglobin, capillary, and mitochondrial content. Type II muscle fibers, on the other hand, have lower aerobic ability, lower levels of myoglobin, less capillaries, and are better suited for anaerobic metabolism. Type IIa fibers have more aerobic potential than type IIx, but less aerobic potential than type I fibers (64). Higher proportion of type I muscle fibers has been significantly associated with higher serum adiponectin concentrations, whereas the proportion of type IIb muscle fibers was inversely associated (65).

The proportions of muscle fiber types vary by race, with more anaerobic (type II) fibers in NH Black people, and more of type I fibers in NH White people. Compared with type II muscle fibers and especially the type IIb (IIx), which are insulin resistant, type I muscle fibers are insulin sensitive (66, 67). Type II myofibers are also associated with obesity, hypertension, and diabetes, particularly in NH Black people (64, 68).

Obesity is a major risk factor for type 2 diabetes. Studies have shown that the proportion of type I muscle fibers correlate inversely and type IIx (IIb) muscle fibers correlate directly with the BMI and percent body fat (67, 69, 70). Obese diabetic individuals have a lower proportion of type I muscle fibers and a higher proportion of type IIx muscle fibers (67, 69, 70). In a study on the prevalence of muscle fiber type in African American Blacks, obese African American women showed a significantly more type IIx (IIb) muscle fibers than lean subjects (67). In addition, obese African American women showed significantly lower percentage of type I muscle fibers and a higher percentage of type IIx fibers than in obese Caucasians (67). These results are consistent with higher obesity prevalence, higher weight gain, and insulin resistance in African American Blacks.

Thus, because of an inherited lower amount of skeletal muscle fiber type I and higher amount of fiber type IIx (IIb), African American Blacks may be genetically predisposed to type 2 diabetes, decreasing oxidative ability and fat oxidation, resulting in increased accumulation of fat in muscular tissue.

Obesity is intimately linked to elevated circulating insulin levels, reduced insulin sensitivity, and insulin resistance (71). High blood glucose and insulin levels with corresponding insulin resistance have been correlated with poor outcomes in breast cancer patients (72–75). Human pulmonary tumor data have shown that cancer cells display increased glucose consumption which may lead to fueling of cancer cells (76). The increased glucose metabolism in cancer cells even in the presence of oxygen is known as the Warburg effect, which is defined by intense lipogenesis, increased aerobic glycolysis and low mitochondrial oxidative phosphorylation capability even in the presence of adequate oxygen (77). High blood glucose levels and hyperinsulinemia, which are frequent in obese individuals, may provide a selective growth advantage to the cancer cells (43).

Biologically, obese persons, and especially obese postmenopausal women, have increased serum levels of estrogens, estrone and estradiol, and decreased levels of sex hormone-binding globulin (SHBG), a glycoprotein which binds estradiol the major female steroid hormone and inhibits its function (78). As a result, this increased levels of the steroid hormones in obese persons may potentially enhance tumor progression and recurrence. Decreased levels of SHBG also increased the levels of circulating androgens which may contribute to tumor progression on their own and to tumor promoting effects by their further conversion to estrogens by adipose tissue (79). In addition, estrogens increase leptin production, which leads to breast cancer cell proliferation and cancer progression. It has been shown that excess adipose tissue contributes to increased circulating insulin and IGF-1 (80) resulting in poorer outcomes.

The impact of obesity on increased likelihood of premenopausal TNBC may partly explain the higher incidence of TNBC among young AA and Hispanic women compared to NH Whites. According to data from the 2013–2014 National Health and Nutrition Examination Survey, the prevalence of overweight or obesity (BMI ≥ 25 kg/m^2^) for women between 20 and 39 years of age was 56.7% for AA, and 33.2% for NH White women (36). If obesity alone is the determinant of ER/PR and HER2 status, then obese Black and non-Black women should have similar proportions of ER/PR and HER2-negative tumors. However, obese Black women have 4-fold more triple-negative tumors than obese non-Black women (28), suggesting other possible mechanisms that influence whole body obesity are crucial in determining ER/PR and HER2 expression.

### Biological Mechanisms of TNBC Risk in AA Women

The 2017 CDC and NCI review of trends in population-based BC incidence and mortality among all racial groups in 2014 showed that although the incidence rates were comparable for AA and NHW American women (122.4/100,000 vs. 124.8/100,000), the mortality disparity was present (Table 4, ref. a). AA patients experience substantially higher breast cancer mortality than NHW women (28.1/100,000 vs. 20.0/100,000) (Table 4, ref. a). Compared to NHW women, 5-year survival was also worse for AA women (91 vs. 80%) (Table 4, ref. b). AA patients were most likely to be diagnosed at stage III (24.1%) with high tumor grade (64.6%) and TNBC (29.6%) (Table 4, ref. c). NHW women had the highest proportion of tumors diagnosed at stage I (45.6%) and ER-positive tumors (76.1%) (81). AA women had significantly fewer HR-positive tumors and significantly more TNBC than NHW women. There was no significant difference in HER2 positive tumors (Table 4, ref. d).

**Table 4:** Disparities in breast cancer risk and survival among African American and Non-Hispanic White women.

**Characteristic**	**African American**	**Non-hispanic white**	***p-*value**
Incidence rate (2014)^a^	122.4/100,000	124.8/100,000	
Mortality rate (2014)^a^	28.1/100,000	20.0/100,000	
Five-year survival 2005–2011(%)^b^	80	91	
High tumor grade (%)^c^	64.6	43	
**Breast cancer subtype**^**d**^			
Hormone receptor positive (%)	42.9	67.3	<0.001
HER2 positive (%)	16.8	17.1	0.96
Triple negative (%)	36.3	13.7	<0.001
**Gene mutations**^**e**^			
*TP53 mutation (%)*	46.3	27.3	<0.001
*MLL3 (%)*	11.6	6.1	0.033
*PIK3CA (%)*	23.1	33.8	0.021
**Proliferation marker**^**f**^			
Ki67 >10%	88	54	<0.001
**Inflammatory cytokines**^**g**^			
IL-6 (pg/ml)	4.51	0.88	
Resistin (ng/ml)	18.8	7.33	

*MLL3, Histone-lysine N-methyltransferase*.

*PIK3CA, Phosphatidylinositol-4,5-bisphosphate 3-kinase*.

*IL-6, Interleukin-6*.

*Ki67, Marker of proliferation*.

*Data from*:

a *US Cancer Statistics: 1999–2014 Incidence and Mortality. Web-based report available at http://www.cdc.gov/uscs*.

b *ACS. Cancer Facts & Figures for African Americans. 2016–2018. Atlanta: ACS, 2016*.

c *Warner et al. (81)*.

d *Keenan et al. (82)*.

e *Ademuyiwa et al. (83)*.

f *Sullivan et al. (84)*.

g *Deshmukh et al. (85)*.

The reasons underlying the racial disparity in breast cancer outcome are multifactorial. Several socioeconomic issues, including income, access to care, and treatment delays, have been involved to play a critical role (86–90). However, many studies have found that the disparity remains even after adjustment for socioeconomic and treatment differences (87, 89, 90). Some studies have suggested that tumor biology may contribute to the inequity (91, 92). Although TNBCs are known to occur more frequently among AA women (26, 92–94), the influence of somatic genomic profiles on breast cancer disparity is still not clear. Somatic mutation analysis revealed racial differences in high prevalence (>5% in the TCGA dataset) genes (*TP53, PIK3CA, MLL3*) in all breast cancer patients, irrespective of clinical subtype (83). *TP53* alterations were observed in 46% of all AA women vs. 27% of all Caucasians; *p* < 0.001, *PIK3CA* alterations: 23% in all AA women vs. 34% in all Caucasians; *p* 0.021, and *MLL3* alterations: 12% in all AA women vs. 6% in all Caucasians; *p* value 0.034 (Table 4, ref. e). *TP53* is a tumor suppressor gene and plays a key role in controlling cell proliferation, cell survival, and genomic integrity (95). Disrupting *TP53* function promotes inappropriate survival leading to uncontrolled proliferation of damaged cells. Olivier et al. (95) have shown that *TP53* mutations were more frequent in breast cancer tumors of ductal and medullar types, aggressive phenotype, and low hormone receptor content. The MLL3 (Histone-lysine N-methyltransferase) gene is tumor suppressor gene because it is often deleted in myeloid leukemia patients (96, 97). Recent studies have reported reduced MLL3 expression in many breast tumors (98). The phosphatidylinositol-3-kinase (*PI3K*) pathway is one of the most frequently enhanced oncogenic pathway in a variety of malignancies (99). In particular, breast cancer tumorigenesis is believed to depend on the PI3K pathway. Many studies have found mutations of the phosphatidylinositol-4,5-bisphosphatase-3-kinase (*PIK3CA*) gene coding for catalytic unit of PI3K mutations to be good prognostic markers in patients with early breast cancer (99).

Differential gene expression of primary breast cancer has discovered intriguing differences between races (14, 100–103). In the overall comparison between primary breast cancer tumors from AA and Caucasian American (CA) women, Steward et al. identified 342 differentially expressed genes and other transcripts (log2 fold-change > 1.0 or < −1.0 and *P* < 0.001) with few directly linked to breast cancer (14). Among 100 genes significantly overexpressed in AA breast tumors, resistin, an adipocytokine that induces insulin resistance and exerts proinflammatory effects (104, 105), showed 2.25 log_2_ fold-change (*P* = 3.05E-06). Breast cancer patients have significantly higher circulating levels of resistin compared to controls (106), and its higher level are associated with poor patient survival and more malignant clinical status (85, 107). Because of the strong link between obesity and cancer mortality, overexpression of resistin suggests important role of this gene in AA breast cancer (108–110). Similarly, a number of genes, including ADAM metallopeptidase with type 1 motif 15 (ADAMTS15: −1.29 log2 fold-change; *P* = 1.82E-04) have decreased expression in AA women. ADAMTS15 is a metalloprotease known to inhibit breast cancer cell migration. Thus, the decreased expression of this gene may have implications for the development and progression of breast cancer in AA women.

### Social Determinants of TNBC Risk in AA Women

There is growing evidence that breast cancer patients with lower socioeconomic status (SES) are more likely to be diagnosed with advanced stages of breast cancer (111–114). The clinical outcomes in AA women with TNBC are worse when compared with European-American women who have the disease. AA patients were more likely to be diagnosed at a younger age, at a more advanced stage of the disease, to have larger tumors, to be unmarried, to live in lower SES neighborhoods, and to have public or no health insurance compared with European-American patients (all Ps < 0.05) (115). A number of other contributing factors may cause the deficiencies in treatment and care among AA breast cancer patients, such as less likely to receive the standard of care (116, 117), financial hardships caused by cancer care (118), need for time taken from work (119), and problems with travel (120, 121), which may also disproportionally affect the cancer treatment and care of AA women. Besides socioeconomic deprivation, survival may also be influenced by patient social context disproportionately affecting AA women, at the individual or neighborhood levels, and social inequality, leading to metabolic dysfunction associated with abdominal obesity (122–124).

In a large study of women diagnosed with invasive breast cancer in California, Tao et al. (115) found that disparities between AA and European-American women in breast cancer mortality varied according to breast cancer molecular subtype and the tumor stage. Within Stages II and III HR+/HER2- breast cancer, they found 31–39% higher rate of breast cancer specific deaths in AA than European-American patients after adjustment for tumor characteristics, first course of treatment, demographic factors, neighborhood SES, and insurance status (115). However, these factors, especially neighborhood SES, fully explained overall mortality differences in Stage I HR+/HER2-, Stage I and II HR+/HER2+, and Stage II TNBC, suggesting that early detection and early diagnosis plays a critical role in efforts to eliminate disparities. This finding is consistent with prior reports of a substantial impact of neighborhood SES on racial disparities in breast cancer mortality (13, 87, 114). Prior studies focusing on TNBC cancer reported that several molecular factors (*see Biological mechanisms of breast cancer risk in AA women)*, as well as epidemiological factors, including reproductive and patient demographic factors (125), differed in prevalence among racial groups, indicating clear association between unequal living standards and increased levels of co-morbid disease.

Using the univariate multinomial logistic regression model, in their studies on associations between sociodemographic, tumor characteristics, and breast cancer subtypes, Llanos and collaborators (126) reported that compared to the luminal A subtype as the referent group, women with TNBCs were more likely to be younger at diagnosis, African American, and of lower SES (less than college educated, and income below the state median of $70,000). Additionally, women with TNBCs were more likely to have tumors that were self-detected, poorly differentiated, higher stage, larger, p53 positive and Ki67 positive and were less likely to have a history of benign breast disease. The findings of this study support associations between sociodemographic and clinicopathological characteristics of tumors, and biomarker status-based BC subtypes, specifically showing that the more aggressive tumor phenotypes were more likely to occur among women who were diagnosed at younger age, African American, and/or of lower SES.

Higher prevalence of the socioeconomic disadvantages experienced by AA women in their communities partially explains the breast cancer outcome disparities observed between AA women and White American women. Recent US Census Bureau reports show that poverty levels in AA women are more than twice as high as in White American populations (25.8 vs. 11.6%) (29). Despite overall drop in uninsured rates for all Americans from 16.0% in 2010 to 11.5% in 2014, disparities in this socioeconomic parameter persist, with 11.9% of the AA population being uninsured compared to 8.2% of the White American population (127). Barriers in healthcare access resulting in diagnostic and treatment delays explain partially variances in BC stage distribution and mortality disparities. Among BC cases regional disease is diagnosed in approximately one-third of the AA patients compared with one-quarter of White American patients, and localized disease is detected in approximately one-half vs. two-thirds, respectively (128).

There is a strong correlation between socio-demographic characteristics, lifestyle, diet, and obesity. Among AA women the prevalence of obesity (BMI≥30 kg/m^2^) is higher (58.6%) compared to non-Hispanic White women (33.4%) (34). Because AA women have higher incidence of obesity, and obesity predicts poor survival, it is speculated that obesity is a potential driver of aggressive TNBC biology in AA women. The mechanistic link between obesity, insulin signaling, and aggressive subtypes of TNBC is increasingly being supported. Tissue inflammation as a result of obesity promotes production of elevated levels of inflammatory cytokines (IL-6, IL-8, TNF-α, and leptin). IL-6 and IL-8 signaling leads to activation of STAT3, NF-κB, and EZH2 signaling pathways and predicts poor prognosis in women with TNBC (129).

**Figure 1:**
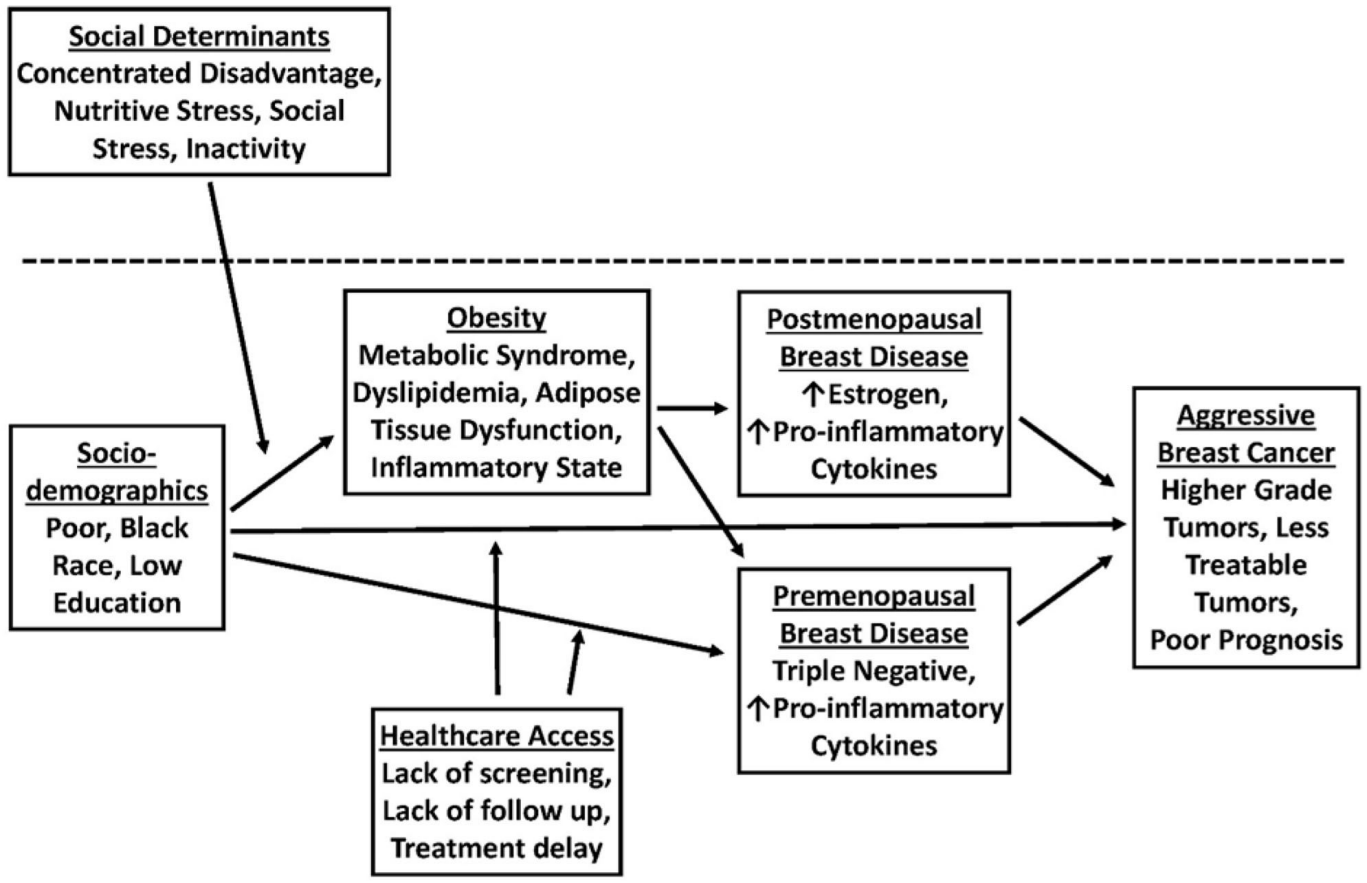
A model for the role of obesity in promoting breast cancer (BC) disparities in African American (AA) vs. Non-Hispanic White (NHW) women.

Additionally, there is also a clear association between unequal standards of living and increased rates of co-morbid disease. Disparities in income, lack of access to fresh vegetables and nutritious food, lack of healthcare access, unsafe neighborhoods, and lack of physical activity can promote co-morbid diseases such as obesity and diabetes, which in turn may drive signaling pathways associated with aggressive biology in TNBC (Figure 1). Obesity and accompanying tissue inflammation increase tissue factors, such as inflammatory cytokines, increased activation of insulin-like growth factor 1 receptor (IGF1R), and increased expression of vascular endothelial growth factor-activated genes that contribute to aggressive breast cancer biology (38). Taken together, it is clear that disparities in physical activity, access to healthy food, and a lack of safe neighborhoods disproportionately promote obesity and poor metabolic health in AA women and aggressive TNBC biology.

## Conclusions and Future Direction

Significant disparities exist between AA and European-American women in the incidence and nature of breast cancer. AA women have twice the odds of being diagnosed with TNBCs than NHW women (Table 1). The reasons underlying the racial disparity in breast cancer outcome are multifactorial. Breast cancer risk factors have been divided into two major categories based on whether they are related to lifestyle (non-biologic) or related to factors unrelated to lifestyle (biologic). The poor prognosis of AA women with breast cancer has been attributed to both biologic and non-biologic factors. TNBC is more common among AA and western sub-Saharan African breast cancer (BC) patients compared with White/Caucasian Americans (WA). In a number of studies, striking similarities in disease epidemiology, risk factors, tumor biology, and genetics were observed between African and AA breast cancer patients, suggesting that West African ancestry is associated with inherited susceptibility for TNBC (24, 130). Further, altered expression levels of several genes associated with cellular growth and differentiation, invasion, and metastasis have been found in breast cancer tumors of AA women to a greater extent in NH White women and are considered to be important contributors to the disparities (14).

Central obesity measures, such as waist circumference (WC) or waist-to-hip ratio (WHR), are associated with an array of hormonal and metabolic changes and may be a better predictor of the risk of premenopausal breast cancer than overall adiposity. Waist circumference or WHR in premenopausal women has been found to be associated with higher levels of insulin-like growth factors or androgen levels, and thus central adiposity may be particularly relevant to premenopausal breast cancer risk (131, 132). Studies have shown that risk of TNBC tumors was reduced for women with a high BMI, but increased for those with central obesity, in particular, AA women (48). These and other findings support the notion that TNBC tumors may be more linked with the components of the metabolic syndrome (central obesity, insulin resistance, decreased tolerance to glucose, dyslipidemia, and hypertension) than by estrogens (133, 134).

A major drawback in performing studies that investigate the potential association between obesity and breast cancer is that the studies are simply driven by anthropomorphic measurements rather than by the metabolic health of the individual, although BMI and WHR likely play different roles in different breast cancer subtypes. The Edmonton Obesity Staging System (EOSS) is a five-stage system of obesity classification (stages 0–4) which is complemented by a clinical staging system that considers the metabolic, physical, and psychological parameters to provide meaningful framework for medical decision-making in optimal obesity treatment and pharmacologic interventions (135). A patient with obesity related risk factors (stage 1) but diagnosed with type 2 diabetes would be categorized as EOSS stage 2 (136). Patients with obesity-related end organ damage or severe disabilities from obesity-related chronic diseases would be classified in higher stages of EOSS (stages 3 and 4) (135). EOSS has been reported to be a better predictor of mortality than BMI or metabolic complications (137). Biological differences in body composition among NHW and AA women modulate risks resulting from obesity and obesity associated increased risk of breast cancer subtypes and comorbidities. EOSS may be useful for clinicians in the identification of breast cancer patients at higher mortality risk, and provide a framework to aid decision making to reduce mortality rates.

While biological differences contribute to breast cancer disparities, it is also generally recognized that social and behavioral factors play a major role in the racial differences observed in breast cancer mortality. Barriers to healthcare access because of low socio-economic status (SES) lead significantly to disparities in the outcome of breast cancer (115). Low SES is associated with higher risk of aggressive premenopausal breast cancer as well as late-stage diagnosis and poorer survival in AA women (13, 130, 138). Chu et al. explained the role of race/ethnicity in overall survival of TNBC patients using the data from a single hospital in Louisiana (139). After controlling for socioeconomic status (SES) and standard of care, they found that the overall survival of TNBC patients were not dependent on race/ethnicity. Chu et al., also reported that in indigent population, race or ethnicity had no impact on ER-negative breast cancer as well as other breast cancer outcomes (140, 141). These results support the idea that access to health care as a potential driver of unequal TNBC survival between AA and CA women. Our recent study on the TNBC cases from the Louisiana Tumor Registry suggested that neighborhood disadvantage (as measured by CDI, concentration disadvantage index) was associated with more advanced stages of TNBC at diagnosis and poorer stage-specific survival (142). Although TNBC incidence was higher in AA, the CDI did not fully explain the disparities, suggesting a role of biological differences.

Although many epidemiological studies rely on self-declared race, we do acknowledge the heterogeneity of individual genetic makeup (genetic admixture). Recent studies have described the limitations of using self-reported race and suggested to use ancestry informative markers to characterize individual's biological ancestry (143–145). Advancement of next generation sequencing should help us to perform genome wide association study to identify genetic factors responsible for health disparities among different racial and ethnic population. It is important to adjust for genetic race and ethnicity in the analyses of genetic susceptibility of diseases, however at present there is no single accepted standard method to characterize race and/or ethnicity (144, 146, 147).

Cancer outcomes are a function of a combination of factors including intrinsic biological factors, modifiable behavioral risk factors and decision-making, as well as characteristics of interactions between the medical system of patients and the health care system itself. Knowing how biological, social, and health-care variables work together to affect outcomes will benefit clinicians, researchers, and policy makers to pave the way to identify more innovative approaches to address breast cancer disparities and how do they combine to determine breast cancer risk.

## Author Contributions

OP conceived the original idea and wrote the manuscript with inputs from FH, DD, AL, and RS. LM provided critical feedback and guidance in the preparation of the manuscript. All authors contributed to the final version of the manuscript and approved it for publication.

## Conflict of Interest

The authors declare that the research was conducted in the absence of any commercial or financial relationships that could be construed as a potential conflict of interest.
